# Optimizing Coaching During Web-Based Relationship Education for Low-Income Couples: Protocol for Precision Medicine Research

**DOI:** 10.2196/33047

**Published:** 2021-11-04

**Authors:** S Gabe Hatch, Diana Lobaina, Brian D Doss

**Affiliations:** 1 Department of Psychology University of Miami Coral Gables, FL United States

**Keywords:** online relationship education, precision medicine, low-income couples, coaching, OurRelationship, ePREP

## Abstract

**Background:**

In-person relationship education classes funded by the federal government tend to experience relatively high attrition rates and have only a limited effect on relationships. In contrast, low-income couples tend to report meaningful gains from web-based relationship education when provided with individualized coach contact. However, little is known about the method and intensity of practitioner contact that a couple requires to complete the web-based program and receive the intended benefit.

**Objective:**

The aim of this study is to use within-group models to create an algorithm to assign future couples to different programs and levels of coach contact, identify the most powerful predictors of treatment adherence and gains in relationship satisfaction *within* 3 different levels of coaching, and examine the most powerful predictors of treatment adherence and gains in relationship satisfaction *among* the 3 levels of coach contact.

**Methods:**

To accomplish these goals, this project intends to use data from a web-based Sequential Multiple Assignment Randomized Trial of the OurRelationship and web-based Prevention and Relationship Enhancement programs, in which the method and type of coach contact were randomly varied across 1248 couples (2496 individuals), with the hope of advancing theory in this area and generating accurate predictions. This study was funded by the US Department of Health and Human Services, Administration for Children and Families (grant number 90PD0309).

**Results:**

Data collection from the Sequential Multiple Assignment Randomized Trial of the OurRelationship and web-based Prevention and Relationship Enhancement Program was completed in October of 2020.

**Conclusions:**

Some of the direct benefits of this study include benefits to social services program administrators, tailoring of more effective relationship education, and effective delivery of evidence- and web-based relationship health interventions.

**International Registered Report Identifier (IRRID):**

DERR1-10.2196/33047

## Introduction

### Background

It is estimated that one-third of marriages in the United States are classified as relationally distressed [1]. Individuals with low-income experience, especially high rates of relationship distress and divorce, report significantly lower marital quality, and experience greater fluctuations in marital quality than high-income earners [2-4]. Low-income couples also have higher levels of alcohol use and infidelity and recent analyses indicate that a meaningful percentage break up during federally funded trials [4-6]. Even when low-income couples have access to free relationship education, they are only able to complete between 10% and 60% of the offered classes in nationwide studies [3,7]. Furthermore, a recent meta-analysis of relationship education for low-income couples revealed that even among the statistically significant findings, the effect sizes were trivial (eg, Cohen *d*=0.06-0.09) [8] and not meeting the widely accepted *small* cutoffs for a meaningful-sized change (ie, Cohen *d*≥0.20) [9]. In the light of these limitations, the authors of this meta-analysis called for innovations in curriculum design, improvements in programmatic elements, and exploration of new ways to sustain participant engagement among low-income couples [8].

### The Law of Attrition and Precision Medicine

Attrition problems are not unique to the field of relationship education and appear to be an inevitable outcome of web-based randomized controlled trials (RCTs) [10]. This has led some to propose the law of attrition [11], which argues that participant dropout is a fundamental methodological challenge inherent in RCTs, which makes investigating the effects of web-based interventions particularly challenging. The law of attrition argues that couples are initially drawn to web-based programs because of their innovative properties (eg, they are brief, can be completed from home, and are inexpensive). However, after registering for the program, reasons that individuals may not continue their participation could include (1) being exposed to conflicting messages about the program, (2) rejecting the program for something better, or (3) leaving the program as the individual was dissatisfied with the services they received [11].

Others have argued that the law of attrition could be enhanced by the inclusion of user characteristics [10]. User characteristics (ie, couple- or individual-level differences) may moderate adherence to the intervention. Some of these differences may include relationship problems, levels of motivation to complete the program, baseline levels of symptomology, the need for anonymity, lack of available resources, or living in a remote location. Simply because of couple-level differences, some percentage of the population is likely to complete and benefit from web-based interventions, and some percentage of the population will not, with most of the population likely falling somewhere in between [12].

Thus, determining who is likely to drop out of the program and who is likely to complete the program at the program outset would be a major benefit to administering effective web-based relationship education. Focusing on completion of the program (and its specific activities) rather than aiming to increase the desired outcome (eg, relationship satisfaction) not only decreases attrition but may also increase the desired behavioral changes [12]. The addition of couple-level differences to the law of attrition suggests that sensitivity to couple-level differences would likely decrease attrition and increase the efficacy of web-based interventions.

Recently, strides have been made in machine learning to mirror these ideas, allowing researchers to predict and compare the predicted treatment effects for couples [13,14]. This approach is often referred to as precision medicine. At the heart of this area of research is the idea that average treatment effects from an RCT may not generalize well to treatment targets (ie, individuals or couples). Although treatment decisions made by humans are typically based on a small number of characteristics (eg, race, ethnicity, or gender), humans are unable to make decisions at the multivariate level without computational resources. The ability to estimate couple-level treatment effects would allow for treatment options that are directly tailored to estimate the unique needs of each unique couple that a practitioner would encounter [13,14].

### A Review of Evidence-Based Relationship Education Programs

This precision medicine and machine learning approach is relatively new to the field of relationship education and web-based health interventions. Thus, starting a precision medicine approach with programs that have already been shown to be evidence-based and beneficial would be worthwhile. Arguably, OurRelationship and web-based Prevention and Relationship Enhancement Program (ePREP) are the 2 web-based relationship education programs with the largest evidence base. OurRelationship and ePREP contain only 6 to 10 hours of content—substantially less content than comprehensive programs previously delivered by the federal government. The OurRelationship program is a web-based, self-help adaptation of integrative behavioral couple therapy that emphasizes acceptance and change [15]. The program includes tailored feedback, filmed examples of couples experiencing relationship distress, and i and activities encouraging couples to be more mindful of their relationship dynamics. Currently, the standard of care includes delivering the program in conjunction with four 15-minute calls with a coach to reinforce the material that is learned throughout the program [15-17]. In a nationwide RCT of 300 couples, this short program led to small- to medium-sized gains (Cohen *d*=0.15-0.69) in key areas of individual and relationship well-being compared with a waitlist control group [16]. Furthermore, couples maintained these effects for at least a year following the intervention [18]. The OurRelationship program appears to benefit couples regardless of income, demographic characteristics, and sexual orientation across a number of outcomes of interest (eg, depression, anxiety, relationship satisfaction, relationship positives, and relationship negatives) [19].

The ePREP program is a web-based adaptation of the Prevention and Relationship Enhancement Program, which emphasizes skill building [20,21]. Accordingly, ePREP introduces a set of healthy communication strategies to reduce conflict in relationships. Some of these strategies include the time-out strategy and the speaker-listener technique, as well as how to maintain fun and friendship in the relationship after the program has ended. In several randomized clinical trials, those that have participated in ePREP have reported small- to medium-sized gains in relationship functioning [21-24]. Follow-up studies have found that the improvements in functioning attributable to ePREP were maintained over a 10- to 12-month follow-up period [22,24]. In addition, those who master the strategies taught in ePREP experience superior improvements in constructive communication and relationship satisfaction [23].

Recently, both OurRelationship and ePREP were compared with one another and to a waitlist control condition in a large Administration for Children and Families (ACF)–funded RCT with low-income couples (N=742) [17]. When delivered in conjunction with four 15-minute coach videoconferences or telephone calls, adherence rates in the OurRelationship and ePREP programs were equal, with 69% of couples completing the assigned material. When compared with the waitlist control condition, those in the OurRelationship and ePREP conditions reported increases in relationship satisfaction (Cohen *d*_OurRelationship_=0.53; Cohen *d*_ePREP_=0.42) and emotional support (Cohen *d*_OurRelationship_=0.46; Cohen *d*_ePREP_=0.36), along with decreases in breakup potential (Cohen *d*_OurRelationship_=−0.53; Cohen *d*_ePREP_=−0.43), communication conflict (Cohen *d*_OurRelationship_=−0.78; Cohen *d*_ePREP_=−0.54), and intimate partner violence (IPV; Cohen *d*_OurRelationship_=−0.10; Cohen *d*_ePREP_=−0.08) [17]. Furthermore, these effects were maintained over a 6-month follow-up period [17]. When the 2 programs were compared, only one significant effect emerged; those in the OurRelationship program experienced greater decreases in communication conflict when compared with those in the ePREP program (Cohen *d*=−0.24) [17]. These findings were later reproduced in an independently collected sample [25].

### Predictors of Treatment Adherence and Gains in Web-Based Interventions: An Inconclusive Science

The literature examining general predictors of adherence and treatment gains in web-based interventions is filled with a mix of inconclusive findings. Regularly, demographic variables have been examined as predictors of adherence to and treatment gains in web-based protocols. Gender and education, for instance, were significant predictors of treatment adherence in some studies but nonsignificant predictors in others [26-32]. Findings on the effects of age, in particular, are difficult to disentangle. Younger age has been associated with better treatment adherence in some studies [28], worse treatment adherence in some others [33,34], and still other studies concluded that age might not be associated with treatment adherence at all [29,31,35]. In a study investigating the OurRelationship program, for instance, identifying as Hispanic predicted better adherence to the program [32]; however, other studies have found no ethnic or racial differences between those who do and do not adhere to the program and their subsequent treatment gains [36,37]. An individual’s level of technical competency has been argued to be a barrier in some scenarios but not in others [31]. Some have found that users who are more familiar with technology are more likely to drop out [38], whereas others argue that technological competency positively predicts program adherence [28].

Equally, the literature examining the role that baseline levels of psychopathology or symptomology play in adherence to web-based protocols usually yields inconsistent findings, even among reviews and meta-analyses. For instance, in a recent meta-analysis of self-guided web-based programs (ie, programs without a coach) for depression, levels of depression did not significantly predict treatment adherence [33]. However, other reviews of adherence to web-based programs for depression anxiety have concluded that lower baseline levels of depressive symptoms are reliable predictors of treatment adherence [28]. Several studies have also found that baseline symptoms of anxiety are significant predictors of treatment adherence [28,33], whereas other studies have concluded the opposite [31]. In addition, although some studies concluded that *lower* levels of depression and anxiety predict better adherence to web-based programs for mood disorders [28], *higher* baseline levels of alcohol use predicted better adherence to web-based alcohol intervention programs [34]. A possible reason for these differing effects is that the direction (or lack) of an effect could be moderated by the treatment being provided. For example, studies that concluded that lower baseline levels of depression and anxiety predict better adherence are investigating web-based treatment protocols for anxiety, depression, and relationship distress [28,32], whereas those that do not are investigating adherence to web-based programs to treat specific phobias and posttraumatic stress disorder [31]. In all, the literature examining the role that baseline levels of symptomology play in adherence to web-based interventions is convoluted and often inconclusive.

Despite the inconsistencies in this area, 2 predictors yield relatively consistent findings. First, much like in-person relationship education, high levels of external stress seem to be an important barrier. Specifically, participants who reported that the treatment was too demanding, who reported greater perceived external barriers, more time constraints, more pressure to complete the program, the presence of a physical illness, a family history of mental illness, and those who reported that school or work got in the way were all less likely to adhere to web-based protocols and reported less improvement [28,30,39,40]. The second consistent predictor was motivation. Higher baseline levels of intrinsic motivation have been shown to predict greater treatment adherence in several studies [28,34,39,40].

### Coaching and Supportive Accountability

Although research suggests that brief web-based relationship education yields promising completion rates and relationship outcomes, one area of interest is not well understood: the type and amount of coaching needed for couples to complete the web-based material. Coaches in the OurRelationship and ePREP programs are usually master’s-level clinicians with a degree in psychology that help reinforce the content taught in web-based curricula using telephone or video chat. The supportive accountability model argues that adherence to web-based interventions is primarily predicted by a couple’s accountability to complete the program, that is, the implicit or explicit expectation that the couple is required to complete the material [12]. Accountability, first and foremost, requires the presence of another human being (ie, a coach). This coach can foster a working alliance with the couple, set expectations for program completion, regularly monitor the couple’s progress, and set goals for them in the future. The supportive accountability model further argues that the effect of accountability on program completion can primarily be moderated via 2 processes: motivation to complete the program and how the communication is being delivered [12].

The lack of motivation to complete the program may prevent a couple from completing the agreed upon material [12]. Ideally, coaches can attempt to increase motivation by increasing the importance of tasks through verbal rewards. For example, having more coach calls or sending more frequent reminders are ways of increasing verbal rewards. However, this can often be a balancing act—too much communication could be perceived as being overbearing but too little as a lack of support.

The method of communication can also serve as a moderator for the effect of accountability on material completion. For example, some couples may enjoy the additional connection video conference meetings that their coach provides, whereas others might find regular email contact to be less intrusive. This variability highlights the need to tailor coaching to meet the needs of specific couples. Thus, identifying the best way to determine the amount of coaching a couple needs as well as the right method of communication is critical to the success of relationship education [12].

### The Effect of Having a Coach on Program Outcomes

The effect of a coach has garnered significant attention in the field of web-based interventions. Several studies of individual interventions have investigated the effect of not having a coach (ie, a stand-alone program), conditional coach support (eg, as-needed calls), some coach support (eg, one phone call for all couples), and full-coach support (eg, several phone calls for all couples). The definitions of coach contact vary widely across studies and have, unfortunately, yielded a series of mixed results. Studies of web-based interventions for both couples and individuals have found that more intensive coach support yields (1) better adherence rates, program completion, and treatment outcomes [32,41]; (2) improvements in program completion and some (but not all) outcomes [37,42]; or (3) some effect on program completion but little effect on treatment outcomes [35,39,43].

Some studies have compared web-based content coupled with coach support with only web-based content. For instance, when completion rates from a no-coach version of the OurRelationship program were compared with those of a trial with full coaching (ie, 4 coach calls), 6.1% of individuals in the no-coach trial completed the program compared with 66.1% in the full-coach version of the program [32]. Furthermore, in a web-based problem-solving intervention for depression and anxiety, those who received scheduled support were likely to complete more of the intervention than those who received no support [42]. Finally, in a web-based treatment for depression, those who received support from a therapist saw similar reductions in depressive symptoms, interpersonal problems, and improvements in quality of life compared with those who completed the web-based program without a therapist [35].

Other studies have varied the intensity and type of coaching that couples and individuals can receive and its effects on program outcomes and adherence. When comparing the OurRelationship program with 4 calls to the OurRelationship program with one call, those who received 4 calls were more likely to complete the program (66% vs 36%, respectively) and saw greater reductions in anxiety symptoms than those who had one call [37]. However, those who received 4 calls reported similar gains in relationship satisfaction and decreases in depressive symptoms compared with the one call group [37]. Furthermore, in a web-based intervention aimed at treating panic disorder that compared scheduled coach calls to on-demand coach calls, individuals who received scheduled coach calls saw larger reductions in panic and anxiety symptoms than those who received on-demand services; however, the 2 conditions saw similar reductions in depressive symptoms and perceived stress [41]. In contrast, in a web-based problem-solving intervention for depression and anxiety, those who received scheduled support did not experience differential program outcomes compared with those who received support on request or received support in the form of nonspecific chat or email [42].

Few studies have investigated whether baseline characteristics moderate the effect of different coaching levels. Of the 20 baseline moderators tested in the OurRelationship program, only 2 significant interactions with coaching level emerged. First, those with higher baseline levels of depressive symptoms were actually less likely to complete the program when receiving 4 coach calls compared with the stand-alone version of the program [32]. Second, those who identified as Hispanic were more likely to complete the program with 4 coach calls than in the stand-alone version. However, race, ethnicity, and household income did not moderate differences in program or treatment gains between one and 4 coach calls [37]. A similar pattern of findings emerged when investigating web-based interventions for individuals with stress and anxiety symptoms [39]. A host of candidate variables (eg, age, gender, level of education, occupation, computer expertise, time perspective, perceived treatment credibility, levels of internal or external motivation, and therapist bond) were examined to determine whether background variables moderated the effect of a coach in adherence to the intervention or treatment benefits; however, none of these baseline characteristics acted as between-group moderators [39].

### This Study: Research Aims and Hypotheses

Given past research, this study has 3 aims. The first aim is to investigate how well we can predict the completion rates and changes in relationship satisfaction. As model evaluation has rarely been used in this area of research, no specific hypotheses have been posited. The second aim of this study is to document the most powerful predictors of treatment adherence and gains in relationship satisfaction within 3 different levels of coaching. More specifically, what are the most powerful predictors of treatment adherence and gains in relationship satisfaction in a full-coach condition (with 4 scheduled 15-minute phone calls), automated coach condition (where couples only receive emails), and a contingent coach condition (where couples only receive scheduled coach calls after displaying a pattern of nonadherence)? Although many of the findings in this area of research are inconclusive, 2 findings are relatively consistent: lower external stress and higher intrinsic motivation both predict treatment adherence and gains. Thus, we hypothesize that, regardless of coach assignment, lower external stress and higher intrinsic motivation will emerge as reliable predictors of better adherence to the web-based program and subsequent treatment gains. In the final aim of the study, we intend to use the information gathered in the first 2 steps to determine the most powerful predictors of between-group differences. More specifically, what baseline characteristics are indicative of a given couple’s adherence or gains in relationship satisfaction to couples assigned to the full-coach program compared with automated coaching, contingent coaching, or the waitlist control condition? As this area of research is relatively underdeveloped, no specific hypotheses will be posited.

## Methods

### Procedure

This study was funded by the US Department of Health and Human Services, ACF (grant number 90PD0309; see Multimedia Appendix 1 for peer reviews). The parent study was registered with ClinicalTrials.gov (NCT02806635). The analyses and project design for this study will be preregistered with the Open Science Framework to promote accountability. Data for this study come from a large (N=1248 couples; N=2496 individuals) web-based RCT with a Sequential Multiple Assignment Randomized Trial design [44]. Sequential Multiple Assignment Randomized Trial designs are experiments that allow researchers to develop adaptive interventions and reassign nonadherent participants [44]. This was done by varying the levels and types of coach contact that a couple receives. In this study, couples were initially assigned to one of five conditions: OurRelationship coach, OurRelationship automated (email only), ePREP coach, ePREP automated (email only), and the waitlist control condition. However, couples initially assigned to the no-coach condition that did not complete scheduled web-based activities for >2 weeks underwent a second randomization: to continue the program without a coach or to be assigned to a coach for the remainder of the program. Those who were randomized to continue without a coach were still allowed to access the program, complete the activities, and were still sent automated reminders. In contrast, those who were randomly reassigned to receive coach contact were emailed by a coach and invited to schedule a call. Couples in this contingent coach condition received up to 3 additional calls (for a maximum of 4 calls) depending on where in the program they were when they stopped making adequate progress. This full variability of possible coaching contact, all of which involved random assignment, will allow us to determine the most powerful predictors of which couples should receive 4 15-minute coach calls, those who should simply receive emails, and those who should receive contingent coach contact after displaying patterns of treatment nonadherence.

### Inclusion and Exclusion Criteria

To be eligible for participation, couples had to live in the United States, be married, engaged, or living together for at least six months, report a household income >200% of the federal poverty line, and be between the ages of 18 and 64 years inclusive. In addition, couples had to agree not to seek help for their relationship for the next 6 months and needed to speak English or Spanish fluently. Couples were excluded if they reported severe IPV within the past 6 months (eg, choking, beating, threatening with a deadly weapon, or forced sex), did not have access to highspeed internet, or had previously participated in an ePREP or OurRelationship program.

### Participants

A total of 226 couples were randomly assigned to the OurRelationship coach condition, 145 couples were assigned to the OurRelationship contingent coach condition, and 145 couples were assigned to the OurRelationship automated (ie, email only) condition. Similarly, 222 couples were assigned to the ePREP coach condition, 143 couples were assigned to the ePREP contingent coach condition, and 143 couples were assigned to the ePREP automated (ie, email only) condition. A total of 224 couples were assigned to the waitlist control condition. In total, 4 individuals from the OurRelationship coach condition, 2 from the OurRelationship contingent coach condition, and 2 from the OurRelationship automated condition asked to discontinue and have their data removed from the study. Furthermore, 3 individuals from the ePREP coach condition, 2 from the ePREP contingent coach condition, and 3 from the ePREP automated condition asked to discontinue and have their data removed from the study.

In total, 47.5% (1178/2480) of the sample identified as male, and 52.5% (1302/2480) of the sample identified as female. The average length of the relationship was 5.74 years (SD 5.18). Most of the participants reported belonging to an opposite-gender relationship (2318/2480, 93.47%), and a smaller percentage were in same-gender relationships (162/2480, 6.53%). Most participants identified as White non-Hispanic (1520/2480, 61.29%), fewer identified as Black (434/2480, 17.5%), White Hispanic (279/2480, 11.25%), Black Hispanic (28/2480, 1.13%), American Indian or Alaskan Native (23/2480, 0.93%), Asian (17/2480, 0.69%), Hawaiian or Pacific Islander (4/2480, 0.16%), and Biracial (86/2480; 3.47%); and 3.59% (89/2480) of participants belonged to a race that was not listed. Furthermore, 6.81% (169/2480) of participants did not have a degree or diploma, 12.3% (305/2480) had earned a general education diploma, 14.23% (353/2480) had completed high school, 8.23% (204/2480) had a technical or vocational certification, 22.54% (559/2480) had some college degree, 8.83% (219/2480) had graduated with an associate’s degree, 10.69% (265/2480) had a bachelor’s degree, and 2.38% (59/2480) had a master’s or advanced degree.

### Measures: Outcome Variables

#### Program Completion

The first outcome of interest in this study is whether a couple completes all the required activities in the program to which they are assigned. Couples that complete all (100%) the activities to which they are assigned will be coded as 1, whereas couples that do not complete all the program activities will be coded as 0.

#### Relationship Satisfaction

Relationship satisfaction will be measured using the Couples Satisfaction Index–4 [45]. The Couples Satisfaction Index was developed using item response theory and has better psychometric properties than much longer measures of relationship quality. The reliability of the current scale is excellent among low-income, help-seeking couples (Cronbach α=.92) [17]. Furthermore, this scale is highly correlated with past measures of relationship quality (*r*>.78) and positive communication (*r*>.75), providing evidence its validity [45].

### Measures: Predictor Variables

#### Demographic Variables

Although support for demographic variables and their relationship to treatment adherence varies, a host of demographic variables will be included as candidate predictor variables. Predictor variables include race, ethnicity, household income, age, gender, as well as identifying as a same-gender couple.

#### Baseline Measures of Relationship and Individual Symptomology

In line with the consistencies in previous research and in addition to relationship satisfaction, several measures of relationship symptomology will be used as possible candidates to predict program completion and gains in relationship satisfaction.

##### Breakup Potential

Breakup potential will be assessed using a three-item Likert-style measure adapted from the Marital Instability Index (*The thought of ending my relationship has crossed my mind*), which has good internal consistency (Cronbach α=.83) in past RCTs involving low-income couples [17,46].

##### Relationship Commitment

To assess relationship commitment, this study plans to use a single-item Likert-style measure developed by the ACF (ie, *How much do you agree or disagree with this statement? I view our marriage/relationship as lifelong*).

##### Intensity of the Biggest Relationship Problem

The intensity of a couple’s biggest relationship problem could be a powerful predictor of nonadherence. This construct was measured using a single item on a Likert-style scale (ie, *How big of a problem is the biggest problem (core issue) in your relationship?*).

##### Communication Conflict

This form of negative communication was measured using a Likert-style scale developed by the ACF (ie, *My partner/spouse was rude or mean to me when we disagreed*). Past RCTs using this measure have reported good internal consistency (Cronbach α=.89) [17].

##### Emotional Support

As another measure of baseline relationship symptomology, emotional support was measured using a five-item measure developed by the ACF. Past RCTs using this measure have reported good internal consistency (Cronbach α=.83) [17].

##### Intimate Partner Violence

Minor levels of IPV were assessed using 7 items created in consultation with the National Domestic Violence Hotline [17]. Among others, participants were asked to indicate how often their partner pushed, slapped, or punched them in the past month. Responses were recorded on a Likert-style scale. Internal consistency in past RCTs with low-income couples has been acceptable (Cronbach α=.78) [17].

#### Measures of External Stress

##### Overview

In addition to including predictors of relationship well-being, the inclusion of measures assessing external stress has been shown to be a consistent predictor of treatment adherence. In addition, as this is measured at the individual level, this may highlight within-relationship differences that may aid in the prediction of treatment nonadherence and satisfaction gains. This study intends to measure psychological distress and general health using the following measures:

##### Psychological Distress

To assess individual distress, this study intends to use the Kessler Psychological Distress Scale (*During the past 30 days, how often have you felt nervous*) [47]. This is a high-precision, 6-item, Likert-style measure developed using the item response theory. In a past RCT with a low-income sample, this scale had good internal consistency (Cronbach α=.86) [48]. Furthermore, this scale has excellent discrimination when attempting to detect severe cases of psychopathology, providing evidence of its validity [47].

##### Perceived Stress

To assess perceived distress, this study intends to use the Perceived Stress Scale, a 4-item Likert-style measure (*In the last 30 days, how often have you felt that you were unable to control the important things in your life?*) [49]. A past RCT has indicated that internal consistency was acceptable (Cronbach α=.74) [48].

##### Chaos

Another measure of external stress will include a single-item measure of chaotic events that an individual may experience in their relationship. This will be assessed using a check-all-that-apply item developed by the ACF; that is, *the following are a list of events that may cause stress for you and your partner. Check each thing (or similar thing) that has happened to you in the past month*. Some of the answers from this item include *changes in work schedule, changes in childcare, changes in family structure, or changes in finances*.

##### General Health

Impacts on the physical well-being (eg, a chronic illness) of one individual may prevent both members of the dyad from completing the program. To assess general health, this study intends to use a single-item measure of general well-being on a Likert-style scale developed by the ACF (ie, *In general, how would you describe your health*).

#### Measures of Motivation

A final category of predictors of treatment adherence is the motivation to complete the treatment. In line with the literature, motivation will be measured using grit and motivation to change.

##### Grit

One measure of motivation is grit or passion and perseverance for long-term goals under challenging circumstances [50]. Despite the challenges accompanying relationship distress, perseverance to complete the program regardless of these challenges may be an important indicator of treatment adherence and gains. In the standardization sample, internal consistency ranged from acceptable to great (Cronbach α=.73-.83), and the measure was strongly correlated with conscientiousness (*r*=.73-.77), providing evidence of the scale’s reliability and validity.

##### Motivation to Change

Participants’ motivation to change will also be measured with the following item adapted for this study: *Which of the following statements best describes your view of your current relationship problems* [51]? Participants will respond on a four-point Likert-style scale ranging from *I don’t think I have relationship problems and therefore nothing should be done about it* (coded as 0) to *I know I have relationship problems, and I am here to take action to work on them now*.

### Data Analysis

In an effort to reduce computational complexity, rather than using individual responses, responses from both members of the couple will be combined in the average score for the couple for each of the continuous predictor variables listed above as well as the continuous outcome variable (ie, relationship satisfaction).

#### Data Analysis Plan for Aim 1: Examining the Prediction Accuracy of the Within-Group Models Using the Random Forest Algorithm

The first research question asks: how well can one predict program completion and anticipated gains within (1) the full-coach condition, (2) the automated coach condition, and (3) the contingent coach condition? To ensure that the different programs do not account for a substantial portion of the variance when predicting program completion or changes in relationship satisfaction, a test will be performed where 2 models will be created and compared. In the first model, all predictors will be interacted with the level of coaching (full, contingent, and automated) and the program assignment (ie, OurRelationship and ePREP) resulting in Predictor×Treatment×Coaching interactions. However, the second model will maintain all predictors by coaching interactions but drop all program assignment interactions (ie, resulting in only Predictor×Coaching interactions). Next, the root mean square error (RMSE) between the 2 models will be compared. If the null hypothesis does not get rejected (H_0_: RMSE_Model 1_ = RMSE_Model 2_), program assignment will be ignored resulting in 3 conditions (full, contingent, and automated coaching), which will be used in the proceeding aims. If the interactions account for a significant portion of the variance (H_a_ = RMSE_Model 1_ ≠ RMSE_Model 2_), independent models will be built by treatment (ie, OurRelationship or ePREP) and coach condition (full, automated, and contingent) resulting in 6 models: OurRelationship full coach, OurRelationship automated coach, OurRelationship contingent coach, ePREP full coach, ePREP automated coach, and ePREP contingent coach, which will be used in the proceeding aims.

**Figure 1:**
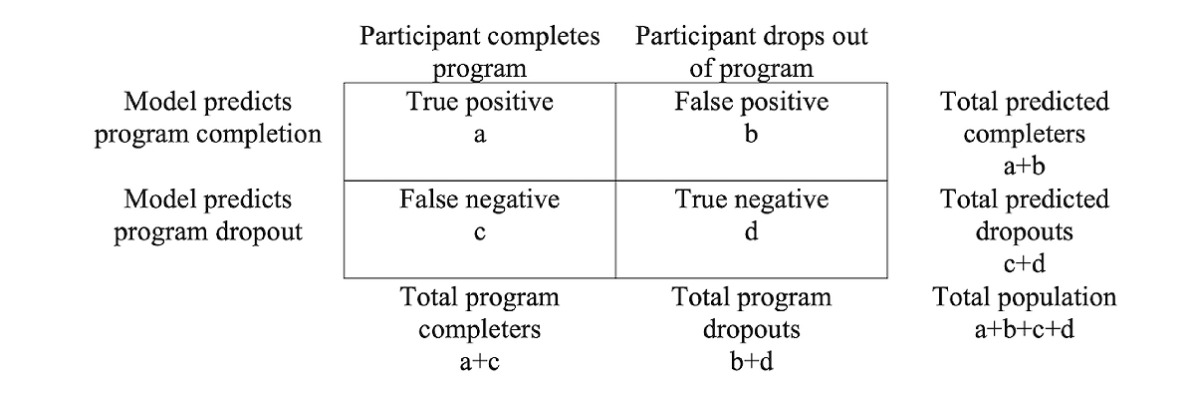
A two-by-two table visually displaying model characteristics.

These models will be built using the random forest algorithm [52]. The random forest algorithm reduces overfitting by bootstrapping or fitting several different trees to subsets of the sample to inform the predictions (ie, bagging) [52]. Once the ensemble of trees is generated, the outputs from all the trees are aggregated, and the prediction is generated. Its ability to predict treatment outcomes for individuals and couples has been proven in several precision medicine studies [13,14]. Thus, in the first aim of this study, the random forest algorithm will be used to accurately predict the within-group likelihood that a couple completes the OurRelationship and ePREP programs in (1) the full-coach condition, (2) the automated coaching condition, and (3) the contingent coach condition as well as the magnitude of their gains in relationship satisfaction. The model characteristics address the model performance on the test data set*.* For binary outcomes (program completion), the model characteristics used to evaluate the validity of the model include sensitivity, specificity, positive predictive value, and negative predictive value of the model built on the test data set (Figure 1). Model sensitivity is measured by the percentage of true positives (ie, a/a+c). Model specificity is the percentage of true negatives (ie, d/d+b). A model’s positive predictive value yields a positive test result and the probability that the participant will complete the program (ie, a/a+b). Finally, a model’s negative predictive value yields negative test results and the probability that the participant will not complete the program (ie, d/d+c). Good models for binary outcomes include models whose sensitivity, specificity, positive predictive value, and negative predictive value are closest to 1. For continuous outcomes (ie, improvements in relationship satisfaction), model accuracy will be evaluated using the RMSE. The RMSE is a measure of absolute fit and is the square root of the variance of the residuals; smaller values indicate better fit.

#### Data Analysis Plan for Aim 2: What Are the Most Important Within-Group Predictors?

The second research question is: what are the most powerful predictors of treatment adherence and gains in relationship satisfaction within (1) the full-coach condition, (2) the automated coach condition, and (3) the contingent coach condition? If, in aim 1, the interaction terms result in a lower RMSE, better sensitivity, or better specificity, models will be constructed based on treatment assignment (ie, OurRelationship or ePREP) and coach assignment (ie, full, contingent, or automated). One statistically powerful way to test the most potent predictors of treatment adherence and gains is through the use of variable importance (VIMP). VIMP is a nonparametric approach within the random forest algorithm, which can help investigators identify which variables play a key role in predicting a binary (eg, program completion) or continuous (eg, improvements in relationship satisfaction) variable [53]. VIMP has been calculated in many ways in the past; however, one way that has shown promise has been through permutation (ie, Briemen–Cutler) importance [53]. This method randomly changes a given variable’s out-of-bag data and compares and averages the permuted prediction error with the original error resulting in VIMP [53]. This process not only helps identify which variables play a key role in prediction but also overcomes issues of overtesting using bootstrapping. Recent studies have used these out-of-bag estimates to generate CIs to test whether a variable has a meaningful effect in predicting an outcome with great success [53]. Indeed, these studies have suggested that 95% CIs that do not include zero will be assumed to have reliable predictors [53]. After identifying the most powerful predictors using VIMP, a multiple regression model will be estimated using the reduced set of variables to aid in the interpretation of the main effects, helping clinicians identify predictors of treatment adherence and gains.

#### Data Analysis Plan for Aim 3: Predicting Between-Group Treatment Outcomes

Assuming that the models from the previous aim have levels of prediction accuracy, each of the within-group models will be used to generate predicted outcomes for each couple’s likelihood of adhering to the program as well as their treatment gains, creating counterfactual estimates of program completion and treatment gains as if each couple in the data set completed each intensity and method of coach contact [14].

To do this, the within-group models built in aim 1 will be used to generate potential outcomes for each couple’s likelihood of adhering as well as their gains in a (1) full coaching, (2) automated coaching, and (3) contingent coach conditions. If, in aim 1, the interaction terms result in a better RMSE, models will be constructed based on treatment assignment (ie, OurRelationship or ePREP) and coach assignment (ie, full, contingent, or automated). The estimates between the conditions for each couple’s between-group treatment differences between the full and automated coaching conditions can be understood as:



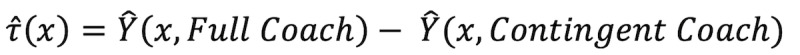



where 
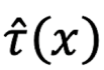
 is the between-group likelihood of treatment adherence or gains for couple x, *Ŷ*(*x, Full Coach*) is the within-group likelihood of treatment adherence or gains for couple x in the full-coach condition, and *Ŷ*(*x, Contingent Coach*) is the within-group likelihood of treatment adherence or gains for couple x in the contingent coach condition [14]. These hypothetical between-group outcomes will then serve as dependent variables in a second random forest to calculate VIMP, which will identify the most powerful predictors of between-group differences. After identifying the most powerful predictors using VIMP, a multiple regression model will be estimated using the reduced set of variables to aid in the interpretation of the main effects. This process will thereby help clinicians determine which couples should be assigned to which level of coach contact.

### Missing Data

Finally, because missing data are anticipated, all missing data will be imputed using the miss-forest algorithm of Ishwaran and Kogalur randomForestSRC package in R (R Foundation for Statistical Computing) [54]. Miss-forest has been shown to robustly impute missing data without overfitting even if the types of data are mixed, there are interactions, or the data are high dimensional [55].

## Results

Data collection was completed in October 2020 and data are being prepared to be analyzed. Overall, 63.8% (286/448) of individuals completed the material in the OurRelationship coach condition, 53.8% (155/288) of couples completed the OurRelationship contingent coach condition, and 54.5% (157/288) of individuals completed the OurRelationship automated (ie, email only) condition. Similarly, 69.4% (306/441) of couples completed the content in the ePREP coach condition, 74.2% (210/283) completed the content in the ePREP contingent coach condition, and 66.2% (188/284) of couples completed content in the ePREP automated (ie, email only) condition. Currently, no other outcomes except for completion rates have been analyzed. Given the large sample size within and between conditions, the current sample is large enough to identify potential predictor variables and evaluate their prediction accuracy. This study is expected to conclude in the summer of 2022.

## Discussion

One of the direct benefits of this study will accrue to social services programs and program administrators of web-based relationship education. The results of this study will allow for more effective tailoring of coach contact to better meet the needs of unique low-income couples experiencing relationship distress. A second benefit is the improvement of web and evidence-based interventions. The federal government is increasing emphasis on delivering evidence-based interventions and, given the social distancing regulations put in place because of COVID-19, participation in web-based programs will likely increase either as an initial intervention or as a backup intervention in cases where in-person services are suspended. Thus, it is important that web-based federal services are as effective and accessible as possible. Overall, this study hopes to help practitioners by generating accurate predictions to match unique couples to the level of web-based programming that will help them to obtain the maximal benefit.

## Appendix

Multimedia Appendix 1 Peer-reviewer report from the Administration for Children & Families (USA).

## Abbreviations

ACF: Administration for Children and Families
ePREP: web-based Prevention and Relationship Enhancement Program
IPV: intimate partner violence
RCT: randomized controlled trial
RMSE: root mean square error
VIMP: variable importance
