# Supplementary material for: Optimizing Coaching During Web-Based Relationship Education for Low-Income Couples: Protocol for Precision Medicine Research
Source: JMIR Res Protoc. 2021 Nov 4;10(11):e33047. doi: 10.2196/33047 (PMC8603166; doi:10.2196/33047)
Supplement: Multimedia Appendix 1 [file resprot_v10i11e33047_app1.pdf]

Applicant Final Panel Summary Report

Average Score: 91.33

Application Number: PD20000296
Application Name: University of Miami
State: FL City: Coral Gables

Criteria Name (Max Score)

1. Relevance to Behavioral Economics/Behavioral Science (25 Points)
2. Background, Significance, and Objectives (25 Points)
3. Research Design and Methodology (20 Points)
4. Qualifications (10 Points)
5. Budget and Budget Justification (10 Points)
6. Management and Dissemination Plans (10 Points)

TOTAL: 100

Scoring Criteria

Criterion 1: Relevance to Behavioral Economics/Behavioral Science

Strength:

Page: 2, 6-10

The applicant provides convincing evidence that the underlying theoretical framework of the study is clearly grounded in a behavioral economics/behavioral science framework. First, the applicant presents two research goals that motivate the use of behavioral economics interventions. The first goal is to determine which types of couples are most likely to benefit by different types of contact for the counseling. The second goal will use information obtained through the variable selection process to create an algorithm used to assign future couples to a method and intensity of practitioner nudges that best addresses their unique needs. Learning about certain characteristics of couples and using this information to match couples with the type of contact that is right for them is consistent with the behavioral economics concept of libertarian paternalism, which the applicant notes is the process of respecting what individuals like while trying to steer individuals into making decisions that will lead to longer, healthier, and better lives. Setting up the choice environment, through the optimal amount of (i.e., providing brief courses) and delivery channel (i.e., providing contact online) for contact in a counseling program is an example of using choice architecture to improve outcomes.

Page: 2-5, 25

The applicant clearly demonstrates how the proposal will use approaches from behavioral economics or behavioral science to address issues facing social services programs and policies and/or issues facing low-income and other vulnerable families in the United States. With regard to the proposal clearly addressing low-income and vulnerable families, the applicant provides convincing evidence that relationship distress is common, prevalent, and disproportionately affects low-income couples in the United States. For example, the applicant cites research indicating that couples in households making less than $25,000 a year are significantly more likely to divorce than couples in households making $25,000-$50,000 or $50K+ and that these couples report significantly lower marital quality. Further, the applicant notes that marital distress also affects the children of low-income couples and cites how marital distress predicts increases in adolescents' emotional and conduct problems. To this end, the sample for the proposed study includes only couples who report a household income less than 200% of the Federal poverty line. With regard to using approaches from behavioral economics to address these issues, the applicant identifies two research goals that draw on concepts related to behavioral economics. Specifically, the applicant plans to change the choice environment by providing counseling that is brief and online, thereby reducing barriers to successful completion of the counseling. Setting up the choice environment by reducing barriers (in this case through reducing scheduling conflicts) is a common technique used in behavioral economics.

Page: 8-10

The applicant clearly situates the project goals and research questions within the context of behavioral economics/behavioral science. The applicant's two primary research goals seek to identify the best way to determine the amount of coaching a couple needs as well as the right method of communication in order to increase the success of relationship education. The applicant notes that relationship education cannot be successful if participants do not comply with the training, and therefore the applicant uses behavioral economics techniques--making the training brief and delivering it online--to increase compliance and completion of the program. The applicant's specific research questions also reflect behavioral economics concepts. The first research question asks what the most important characteristics of the individual or couple are that determine the intensity and method of "nudge" that they will require to complete and benefit from the coaching program. The second research question asks how well, after accounting for all the variables identified in the first research question, one can predict program completion and resulting gains. This information can then be used to match the right low-income couple to the right type and intensity of coaching nudges with the goal of increasing the benefits of the coaching.

Weakness:

Page: 2, 6-10

Although the applicant makes use of approaches from behavioral economics, namely making the coaching brief and online, there are other, more innovative techniques the applicant could have chosen to advance the field's understanding of the efficacy of behavioral economics techniques in an online coaching environment. For example, the concept of implementation intentions, which encourages individuals to explicitly create a plan they will use to accomplish a task or a goal, would be an interesting application of behavioral economics to a new problem--completing online relationship coaching. The applicant could have drawn more from the many behavioral economics innovations in the field that go beyond simply reducing barriers to completion or engagement.

Page: 3-13

As the proposal notes, factors besides behavioral economics (such as cost) also inform the shift to briefer, online relationship education programs; consideration of cost factors is also important.

Page: 64

Scholar proposes two research questions around matching couple's characteristics to nudges, and tying those matches to completion, but does not engage with broader literature around other behavioral levers that can influence engagement and completion (e.g., plan-making, social influence, endowed progress).

Page: 67

Scholar proposes developing a matching algorithm to deliver the appropriate channel and frequency of reminder to promote course completion but does not engage other behavioral concepts such as the effects of framing or implementation intentions.

Page: 9

The application notes that motivation is an important factor in attrition (and one that this study can measure). The application fails, however, to consider how more potentially relevant behavioral concepts and interventions such as implementation intention prompts, social influence and social proof, reciprocity, identity priming, and commitment devices could strengthen the study.

Criterion 2: Background, Significance, and Objectives

Strength:

Page: 13-15

The applicant soundly describes the theoretical framework, research goals, and/or research questions in a way that is clear and logically connected. The applicant first describes the theoretical framework underlying the OurRelationship and ePrep programs, highlighting how relationship distress is hypothesized to develop and the standard of care within each program (both deliver the program online in conjunction with 4 15-minute calls from a counselor). Next the applicant describes the hypothesized barriers to adherence in the programs, noting how the Supportive Accountability Model argues that motivation to complete the program and matching the right method and intensity of practitioner contact to meet the unique needs of the couple will likely lead to increases in program adherence and treatment gains. This provides the motivation for the applicant's research goals, which focus on matching the right method and intensity of the contact by identifying participant characteristics and matching those to the type of intervention participants receive.

Page: 13-16

The applicant clearly provides a comprehensive review of the current literature with reference citations that are complete and consistent with a generally accepted citation format, delineates gaps in the existing literature, and strongly supports the need and objectives for the study. The applicant thoroughly describes the previous research related to the OurRelationship and ePrep programs as well as the literature associated with the concept of treatment adherence and the Supportive Accountability Model. In doing so, the applicant identifies gaps in the literature, noting that although the research suggests that making relationship education briefer and online accessible yields higher completion rates and improved relationship outcomes, one nudge of interest is not well understood: the type and amount of coaching. The applicant describes the concept of the Supportive Accountability Model, and specifically, how it argues that motivation to complete the program and matching the right method and intensity of practitioner contact to meet the unique needs of the couple will likely lead to increases in program adherence and treatment gains. The applicant then reviews the literature associated with program adherence and the characteristics hypothesized to be predictors of adherence, including baseline levels of external stress and symptomology, as well as motivation to complete the program. Importantly, the applicant notes that the literature examining the predictors of treatment adherence to web-based interventions also reports a series of mixed and convoluted findings. The proposed research seeks to help clarify some of these findings using machine learning. Specifically, the applicant argues that inconsistent findings could be due to several factors, but that one explanation consistent with the Social Accountability model is that different predictors of treatment adherence would belong to different types and methods of coach contact. The applicant says that one gap in the current literature is that no study has examined whether predictors of treatment adherence (and subsequent treatment gains) differ depending on the method and type of coaching nudges that a couple may receive. The proposed project seeks to fill that gap.

Page: 14-19

The proposal details that relationship education programs have impacts on outcomes such as self-reported relationship quality, but experience attrition. The proposal also reviews past research on predictors of attrition such as external barriers (for example, child care) and pre-existing motivation.

Page: 18-19

The applicant clearly includes research questions that fill a gap in the literature. The applicant first notes that a nudge requiring additional attention is the method and intensity of practitioner contact that a couple should receive to complete the program and receive the intended program benefit. Additionally, the applicant identifies that no study of which we are aware has examined whether predictors of treatment adherence (and subsequent treatment gains) differ depending on the method and type of coaching nudges that a couple may receive. The applicant's two research questions seek to fill this gap. The first research question will help the applicant understand which characteristics of couples determine the intensity and method of "nudge" they will require to complete and benefit from the OurRelationship program. The second research question will use the findings from the first question to determine how well one can predict program completion and resulting gains. The applicant then notes that this information will be used to match the right low-income couple to the right type and intensity of coaching nudges. However, the applicant argues that addressing these gaps in the literature requires that the method and intensity of coach contact must be experimentally manipulated to create significant variability in coaching. The applicant's research methodology provides this variability.

Page: 2-17

The applicant adequately describes the theoretical framework, research goals, and/or research questions in a way that is clear and logically connected. For example, the proposal thoroughly details the theoretical frameworks of the web-based relationship education programs. The proposal as a whole also documents that relationship education programs experience attrition, how this may reduce their impacts, how the web-based programs may be more effective as a result of reduced attrition, how different nudges may help keep participants engaged in the programs, and how predicting which individuals would benefit from which nudges in order to increase completion of the program might improve the impacts and cost-effectiveness of these programs.

Page: 21-22

The applicant thoroughly reflects a sound description of the anticipated outcomes and benefits of the project, including: (a) the hypothesized results of the study; (b) anticipated outcomes discussed in a way that reflects a solid understanding of critical issues, information needs, and research issues relevant to behavioral approaches and interventions for social services programs serving low-income children, adults, and families; and (c) a thorough description of the significance and implications for policymakers, program administrators, and social services programs. First, the applicant provides hypothesized results for the study, including 1) regardless of the type of intensity or method of coaching nudges, baseline levels of external stress, symptomology, and motivation will all be reliable predictors of treatment adherence, 2) those who experience greater distress will report higher adherence rates as the program becomes more automated but less tailored while those with higher baseline levels of symptomology will experience greater adherence with higher levels of tailoring and less automation, and 3) predictors will vary depending on the method and intensity of coach contact. Next, the applicant clearly discusses how the anticipated outcomes will address critical issues relevant to behavioral approaches and interventions for social services programs by describing how the algorithm developed during the project will be disseminated to program coaches in the form of a reproducible R Script which will assist coaches in assigning couples to a method and intensity of coaching nudges that will best meet the needs of each unique couple. Finally, the applicant clearly describes the significance and implications of the research with two main points: 1) the results will allow program administrators to provide cheaper, simpler, but far more effective services to low-income couples by only providing coaches to couples who are most likely to benefit from them; and 2) identifying the right nudges for the right couples at the right time will increase the effectiveness of programming and counter concerns about the validity of relationship education.

Page: 47

The applicant articulates the relevance of the research questions to the target population of people living with low incomes and helpfully narrows the question to a relevant subpopulation of those who seek out assistance.

Page: 68

The applicant clarifies the policy relevance of the research: creating better matches allows for effective and efficient distribution of limited resources.

Weakness:

Page: 15-22

The applicant plans to measure the outcomes of the intervention primarily as they relate to completion of the program. Although the applicant does measure a host of factors that might relate to program completion, there are few measures on the outcome side that measure whether there were "associated gains" resulting from completion. The applicant could plan to measure outcomes at a period a few months after completion to help identify whether the interventions (brief, online) may help with completion rates but potentially hinder actual "associated outcomes" such as actual improvements in relationships.

Page: 2-24

The applicant does not provide a thorough description of the significance and implications for policymakers, program administrators, and social services programs. The application does not discuss if the available outcome measures of relationship satisfaction, measured at 6 months (4 months after treatment), are policy significant. Therefore, it is unclear why better understanding predictors of program completion, conditional on the type of nudge intervention, is policy significant. In other words, it is not clear how improving the cost-effectiveness of a relationship education program model (for example, through better targeting of nudge interventions to increase program completion) is policy significant if there are no quantified, valued benefits of completing this particular program model (for example, 36-month relationship status, intimate partner violence, or child outcomes). For now, the proposal is limited in that it only demonstrates the significance of this study to program administrators and social service programs (as these programs currently exist), even as the proposal does not discuss any evidence that these programs have policy-significant impacts.

Page: 50

Given the high cost and small effect sizes of relationship education programs, the scholar could broaden his consideration of cost-effectiveness by placing it in the context of literature on alternatives to these interventions (e.g. simply distributing cash in the amount of the program costs to families may reduce stressors and increase well-being more effectively than counseling according to unconditional cash transfer literature).

Page: 53

In examining the behavioral science literature related to choice architecture and program uptake/completion, scholar does not fully engage with the breadth of relevant topics (e.g. plan-making / implementation intentions, social influence / social pressure, micro-incentives / non-monetary incentives or other motivational boosts, etc.).

Criterion 3: Research Design and Methodology

Strength:

Page: 14-17, 24-25

The applicant presents a proposed sample size that is sufficient to answer the range of proposed research questions for the study. For example, the study will use data from 1,250 couples/2,500 individuals distributed across seven research arms, resulting in between 143 and 228 couples per arm and a reported statistical power to detect effect sizes (0.13-0.17) that are smaller than those reported for previous studies of similar interventions in the proposal's theoretical framework discussion.

Page: 20

The applicant presents a research design and methodology that are clearly described; sufficient for addressing the goals of the project; and appropriately link research issues, questions, variables, data sources, samples, and analyses. First the applicant describes the SMART design and how this methodology will allow the applicant to optimize the proposed web-based interventions to match the right nudges to the right couples. This methodology allows researchers to develop adaptive interventions and reassign non-adherent participants, which is critical for the applicant's research goals and questions. The applicant thoroughly walks through the process by which the SMART design was used for the proposed research project, initially assigning and reassigning couples to hone in on the right treatment for each couple, which is the ultimate goal of the project. The applicant describes how the SMART design provides the full variability of possible coaching nudges - all of which involved random assignment - which will allow the applicant to determine the most powerful predictors of which couples should receive four 15-minute coach calls, those who should simply receive emails, and those who should receive contingent coach contact after displaying patterns of treatment non-adherence. The applicant describes outcome measures that will provide information to understand which factors impact adherence as well as which contact approach is most successful for couples with these characteristics.

Page: 24

The applicant clearly describes how the applicant will gain access to the necessary organizations, participants, and data sources required for the project. Specifically, the applicant states that the data for this study come from the evaluation of online services provided by a current Healthy Marriage grantee funded by ACF, and that all the low-income couples that will be included in the current study were seeking help for their relationship online. Given the current grant in place, the participants for this study are easily accessible.

Page: 26-27

The applicant clearly describes the measures that will be taken to ensure adequate protection of human subjects, the confidentiality of data, and consent procedures, as appropriate. First, the applicant notes that before collecting any data, permission was sought and granted from the applicant's institution. With regard to confidentiality of data, the applicant describes the following measures that will be/have been taken: a) the website software is written in PHP and will be deployed on a professionally maintained and up to date FreeBSD server utilizing an advanced virtualization feature of FreeBSD called jails which completely isolates the server software and data from all other users of the system; b) data will be downloaded from the Qualtrics survey using a secure, HTTPS connection and saved directly onto password-protected department servers at the applicant's institution; and c) all electronic files are stored on a lab-specific folder on servers housed in the applicant's institution's Department of Psychology. With regard to the informed consent process, the applicant describes the process, which includes: a) only allowing potential participants to complete the survey after agreeing to and completing the consent document; b) on an initial 15-minute phone call or videoconference with a coach, the coach briefly reviewed the components of the online program, answered any questions the couple had, and reviewed the informed consent form using a script approved by the IRB; c) couples were then given a chance to ask any questions they had about the research requirements and drop out of the study if they so desired without penalty and were asked to verbally agree to key requirements of the study.

Page: 28-33

The applicant clearly and thoroughly describes the measures and their psychometric properties (where possible) to be used for the planned research, and demonstrates that the measures are appropriate and sufficient for the questions and the population to be studied. In addition to describing one key measure, program completion, the applicant identifies several other outcome variables of interest and provides psychometric properties of the measures that are not single questions (many developed for this project). These measures include, the Couples Satisfaction Index (relationship satisfaction), several candidate predictor variables (using Likert-style measures, some developed by the ACF), which clearly link to the first goal of the study, measures of external stress (e.g., Kessler Psychological Distress Scale), and questions related to motivation, such as grit.

Page: 33-34

The applicant presents a clear discussion related to the sensitivity to technical, logistical, and ethical issues that may arise. First, the applicant notes that recruitment for the current project is going to be finished in the coming months. Thus, many of the issues that one may commonly encounter such as obtaining permission from the human-subjects committee, issues related to coach adherence to the web-based program, and recruitment for the study have already been addressed. However, the applicant describes several issues that may require attention during the course of the research. These include: a) protection of participants' confidential data, which the applicant will address by partnering with companies that have advanced security measures in place and making all of the data password protected; b) replication as an ethical problem within the social and biological sciences, which the applicant will address by preregistering the study with the Open Science Framework and published preprints to disseminate the work quickly; c) potentially overfitting the statistical model, which the applicant will address through selecting a sophisticated set of data analyses and taking an additional machine learning course to acquire more information about the process.

Page: 34-36

The applicant provides evidence of a data analysis plan that is clearly and adequately described and incorporates techniques that are appropriate for the specific research question(s) under consideration and for the types of data to be analyzed. For example, the applicant's first research question--Finding the Best Set of Predictor Variables--will be determined by using stochastic search variable selection (SSVS). This will allow the applicant to identify a set of candidate predictor variables with high prediction accuracy while controlling for the uncertainty related to any other candidate predictor variables under consideration. With regard to the second research question--Finding the Model with the Highest Prediction Accuracy--the applicant will attempt to determine how well can one predict program completion and outcomes associated with completion. The applicant describes how this will be accomplished through machine learning, followed by a second step that will compare different machine learning algorithms. Finally, the applicant will evaluate the model's performance using cross-validation and the evaluation of model characteristics (which are all described in the application).

Page: 79

The plan to create predictor variables from training data (a subset of the overall dataset) which can be validated with the remainder of the dataset is a reasonable approach to answer the research questions.

Weakness:

Page: 20, 34-37

The proposal's research design and methodology are insufficient in one aspect as it relates to sample. In order to participate, couples had to have access to high-speed internet. The validity of the predictions may be limited to couples sharing key characteristics of the people whose data comprise the dataset (e.g. help-seeking, digitally literate, able to afford high-speed internet). It would be helpful to have more discussion and estimates from the available literature on low-income access to high-speed internet to gauge how this restriction affects how the study's results may generalize.

Page: 74

The lack of native language or English language proficiency as a demographic variable may limit the ability to assess generalizability of the findings for this study.

Criterion 4: Qualifications

Strength:

Page: 46-47

The proposal demonstrates that the student possesses the research expertise necessary to conduct the study as demonstrated in the application, as well as in information contained in their biographical sketches and/or curriculum vitae, including relevant background, experience, and training on related research or similar projects. For example, the student has previously worked on randomized controlled trials, conducted predictive modeling research, and has clinical experience with relationship education programs. The student expects to propose his dissertation in August 2020, before the grant would be awarded, as required by the rules of this funding mechanism.

Page: 48

The proposal demonstrates that the Principal Investigator (PI) possesses the research expertise necessary to advise the study as demonstrated in the application, as well as in information contained in their biographical sketches and/or curriculum vitae, including relevant background, experience, and training on related research or similar projects. For example, the PI has mentored previous ACF grant-funded dissertations in the relevant content area of Family Strengthening.

Page: 48-49

The proposal demonstrates that the Principal Investigator (PI) has earned a doctorate or equivalent in a relevant field, conducts research as a primary professional responsibility, and has published or has been accepted for publication in at least one major peer-reviewed research journal as a first or second author. For example, the PI holds a Ph.D. in Clinical Psychology, led ACF and NIH-funded research projects, and published first-authored research in journals such as Journal of Consulting and Clinical Psychology.

Page: all

The application is complete and comprehensive. It is clearly written and organized, provides appropriate details, and has sections that are labeled with appropriate headers and sub-headers. There are many aspects related to the various theories and literatures from which the project proposal draws, and the applicant does a good job organizing the previous research and motivating the current research in a way that it understandable.

Page: mentor letter, bio sketches, CVs

The applicant describes how, during his undergraduate degree, he procured several small internal grants which allowed him to conduct and publish two multi-site randomized controlled trials with over 500 participants investigating the impact of sending text message nudges to romantically-involved individuals. The subject matter, delivery vehicle, and methodology explored in these studies all demonstrate extremely relevant experience related to the proposed study. In addition, the mentor notes that the applicant has been involved in every phase of a larger, ongoing ACF-funded project testing the effects of the ePREP and OurRelationship programs for low-income couples nationwide. Most importantly for the proposed project, the applicant has experience serving as both a coach and Coach Supervisor for both the OurRelationship and ePREP programs. In these roles, he conducts calls with couples in the programs and provides supervision to other coaches, and these experiences will give him first-knowledge about the "nudges" he proposes to investigate in this project. The applicant also has taken a central role in designing and implementing research studies examining the impact of these services. In this role, he helped select appropriate measures, set up the online data collection, and checks data as it comes in. With regard to the mentor, the mentor's research over the past decade has been focused on the effective implementation of web-based interventions, including the development of the OurRelationship program, and has served as the primary supervisor of several large randomized controlled trials.

Weakness: None

Criterion 5: Budget and Budget Justification

Strength:

Page: 43-45

Funding is allocated toward the student's time necessary for completing the research and for conference travel and journal submission (dissemination) costs.

Page: 43-45

There is clear evidence that adequate funds have been requested for both the applicant and the mentor to attend two required meetings. These funds are identified as coming from the grant in the case of the applicant and matching funds in the case of the mentor. In addition, funds are budgeted to support travel for the applicant to attend and present the project findings at the National Council on Family Relations in Year 2.

Page: 43-46

The proposal sufficiently includes the commitment for non-federal resources that will contribute to the overall success of the project. For example, the applicant's institution has agreed to fully waive its standard indirect costs rate for this project to allow the applicant to maximize his time and effort on the proposed project. As such, the applicant's institution will provide the applicant with space, materials, and equipment necessary to complete the proposed project which would be valued at $9,206 in Year 1 and $12,625 in Year 2 for a total of $21,885.

Page: 43-46

As evidenced in the project timeline and milestones, the project is expected to be completed in approximately two years. The budget requests the applicant details are justified according to this timeframe and the needs of the proposed project. For example, there is money budgeted to support 33% of the applicant's time for the first academic year and 100% of the applicant's time for the second year. University fees for both years are reflected in the budget proposal.

Weakness:

Page: 45-46

There is insufficient explanation for why the student is unable to access sufficient computing resources through what the university makes available to its graduate students (for example, conducting analyses using university-purchased, remotely-accessible-to-students computing resources) vs purchase of a new personal computer.

Criterion 6: Management and Dissemination Plans

Strength:

Page: 37

There is evidence that the management plan is sound, workable, and illustrates how the project will be carried out, as well as how the student and the mentor will ensure quality control during the project. For example, the applicant provides a proposed timeline for meeting key project milestones including when the entire project is expected to be finished, and when the findings will be disseminated. The applicant also describes that, in order to ensure quality control, the applicant and the mentor will continue to communicate regularly via email and meet multiple times a week to ensure the timely progress of the research project.

Page: 37

The management plan emphasizes frequent communication between the student and mentor and the fact that the student and mentor have successfully worked on numerous research projects in recent years.

Page: 38-39

The applicant includes a feasible timeline for when key project milestones are expected to be met, including when the entire project is expected to be finished, and when findings will be disseminated to a wider audience. The applicant provides a comprehensive table detailing key milestones in the project, including preparing for various conferences and submitting a manuscript detailing the findings to an academic journal. All of the milestones seem feasible in terms of when the applicant expects to complete them.

Page: 38-39

The proposal's dissemination plan thoroughly describes how the student will disseminate the proposed research in ways that are intended to be useful to the broader field, with work plans/timelines that reflect sufficient time and effort for dissemination activities. For example, the plan includes adequate time for preparing and submitting sufficient content for a field-relevant conference such as the National Council on Family Relations (which includes researchers and practitioners).

Page: 38-42

The proposal's dissemination plan is to translate and disseminate the proposed research via a variety of dissemination activities accessible to a wide range of researchers, policymakers, and practitioners.to various audiences (researchers, policymakers, and practitioners). For example, the plan includes conferences, a journal article, publicly available algorithms, and a blog post.

Page: 39-42

The applicant describes how the machine learning algorithms developed for the study will be provided to practitioners through a classification tree, which the applicant shows an example of. The applicant also plans to reach practitioners by attending RECS and presenting the findings, the classification trees, and the correct interpretation of the trees and the associated estimates. The applicant plans to reach the academic community through conferences, manuscripts, and open science journals. Finally, the applicant plans to disseminate the results to policy-makers through attendance at the required conferences, writing research briefings in a timely and proficient manner, updating the project website to include study results with an associated blog, and the mentor disseminating the findings through his networks, such as OPRE, ACF, and the Mathematica staff who are centrally involved in ACF's Healthy Marriage initiative. These dissemination activities (other than mentor networks) are clearly identified in the project timeline with reasonable time and effort being spent on them.

Page: mentor letter

The mentor clearly describes how he will mentor the student, including how often meetings between the mentor and the student will occur, as well as how the mentor will supervise the student's completion of the research and any consultation/collaboration with program partners or policymakers. For example, the mentor and applicant have a standing, hour-long weekly individual meeting to discuss ongoing research activities. They will use these meetings to check in on the progress the applicant has made in the past week and make a plan for the following week. Depending on the week, they will use these meetings to review the mentor's comments on the applicant's written products, discuss/problem-solve any difficulties with ongoing projects, and make a plan for specific tasks in the upcoming week. The mentor describes how, utilizing the table of planned activities included in the project application, the applicant and the mentor will meet at the beginning of the funding period to prioritize those activities in the applicant's semester/summer calendar of planned activities. Then, in their weekly meetings, the mentor and applicant will monitor progress and problem-solve / re-prioritize as necessary.

Weakness: None
